# Small-molecule induction of Aβ-42 peptide production in human cerebral organoids to model Alzheimer's disease associated phenotypes

**DOI:** 10.1371/journal.pone.0209150

**Published:** 2018-12-17

**Authors:** Serena Pavoni, Rafika Jarray, Ferid Nassor, Anne-Cécile Guyot, Steve Cottin, Jessica Rontard, Jacqueline Mikol, Aloïse Mabondzo, Jean-Philippe Deslys, Frank Yates

**Affiliations:** 1 Service d’Etude des Prions et Infections Atypiques (SEPIA), Institut François Jacob, CEA, Université Paris-Saclay, Fontenay-aux-Roses, France; 2 Sup'Biotech, Villejuif, France; 3 Laboratory of Drug Metabolism Research (LEMM), Institute of Biology and Technology Saclay (IBITECS), CEA, Université Paris-Saclay, Gif-sur-Yvette, France; Nathan S Kline Institute, UNITED STATES

## Abstract

Human mini-brains (MB) are cerebral organoids that recapitulate in part the complexity of the human brain in a unique three-dimensional in vitro model, yielding discrete brain regions reminiscent of the cerebral cortex. Specific proteins linked to neurodegenerative disorders are physiologically expressed in MBs, such as APP-derived amyloids (Aβ), whose physiological and pathological roles and interactions with other proteins are not well established in humans. Here, we demonstrate that neuroectodermal organoids can be used to study the Aβ accumulation implicated in Alzheimer’s disease (AD). To enhance the process of protein secretion and accumulation, we adopted a chemical strategy of induction to modulate post-translational pathways of APP using an Amyloid-β Forty-Two Inducer named Aftin-5. Secreted, soluble Aβ fragment concentrations were analyzed in MB-conditioned media. An increase in the Aβ_42_ fragment secretion was observed as was an increased Aβ_42_/Aβ_40_ ratio after drug treatment, which is consistent with the pathological-like phenotypes described in vivo in transgenic animal models and in vitro in induced pluripotent stem cell-derived neural cultures obtained from AD patients. Notably in this context we observe time-dependent Aβ accumulation, which differs from protein accumulation occurring after treatment. We show that mini-brains obtained from a non-AD control cell line are responsive to chemical compound induction, producing a shift of physiological Aβ concentrations, suggesting that this model can be used to identify environmental agents that may initiate the cascade of events ultimately leading to sporadic AD. Increases in both Aβ oligomers and their target, the cellular prion protein (PrP^C^), support the possibility of using MBs to further understand the pathophysiological role that underlies their interaction in a human model. Finally, the potential application of MBs for modeling age-associated phenotypes and the study of neurological disorders is confirmed.

## Introduction

Although much effort has been made to establish in vitro and in vivo models capable of recapitulating pathological AD phenotypes to study pathophysiological mechanisms and test drug candidates [1], more than 99% of drug candidates fail in clinical trials [2]. It has been shown that APP-derived Aβ_42_ oligomers are neurotoxic in vivo in mice [3] and that increasing Aβ_42_ fosters an oligomerization reaction [4] that eventually leads to amyloid plaque deposition. However, the failure at the clinical level of anti-β-amyloid strategies initially validated in genetic mouse models (familial Alzheimer’s disease (FAD) mutations) illustrates the complexity of this human pathology and the difficulty of reproducing all its pathological hallmarks [5,6]. In practice, no animal model appears to develop true AD; a critical need exists for better experimental models that can provide more predictive and physiologically relevant results reflecting the complexity of human brain tissue. [7].

Recently, in vitro models based on iPSC technology have made it possible to study patient-specific phenotypes. In particular, patient-derived neurons can be used to investigate pathological markers and pathways implicated in neurological disorders in vitro [8,9]. This provides an opportunity to overcome limitations linked to the inaccessibility of brain samples and may help to highlight unknown mechanisms and to discover and validate new therapeutic strategies. Two-dimensional (2D) cell culture models of familial (FAD) [10] and sporadic AD (SAD) [11] obtained with iPSC-derived patients' neurons have been shown to reproduce many of the pathological features that characterize AD pathology, notably extracellular or intracellular soluble Aβ accumulation or insoluble Aβ aggregation [8,10,12]. Human iPSC-derived AD neurons, at least in FAD, can successfully recapitulate the hallmarks of early stage of AD pathogenesis [11], but 2D cultures do not allow robust extracellular ß-amyloid aggregation. Indeed, even with the most aggressive AD familial mutations, low levels of Aß species are produced, and the lack of interstitial compartment in 2D cultures prevents ß-amyloid aggregation [13]. Moreover, these 2D models are not able to recapitulate in vivo-like cytoarchitectural organization and the brain’s synaptic connections [14].

Breakthroughs in three-dimensional (3D) cell culture systems have been proposed as a way to more closely recapitulate in vivo CNS architecture: more realistic 3D models could fulfill the existing gap between 2D cell culture and animal models as 3D cerebral organoids can be cultured in the long term and provide a microenvironment more prone to Aß deposition and aggregation in high concentrations [13]. They may better predict in vivo functions and phenotypes [13] through specific gene and protein expression, cell-to-cell and cell-ECM (Extra-Cellular Matrix) network interactions, resulting in higher spatial and chemical complexity [15]. However, several potential difficulties have been identified in studying disorders that require significant maturation and aging [16]. Most notably, the persistent embryonic nature of iPS cell derivatives represents a major hurdle and could compromise their use in modeling age-related neurodegenerative diseases. A variety of protocols are available for differentiating iPSCs into 3D neural cell aggregates, which provide the potential for neurodegenerative disease modeling. However, the lack of cytoarchitectural organization or cellular diversity in these 3D models impedes the recapitulation of complex brain development processes that are dependent on cell-cell interactions across different brain regions. Recently, more complex 3D arrangements of cells resembling human brain tissue, termed brain organoids, which use pluripotent stem cells derived from FAD patients (harboring amyloid precursor protein–APP-duplication or presenilin1 –PSEN1-mutation), have shown the spontaneous emergence of hallmark AD pathologies such as increases in Aβ_42_ soluble fragment secretion and amyloid aggregation, when compared to control organoids [17,18]. However, Alzheimer-related phenotypes obtained in these studies with 3D culture models [18] can vary from those observed in 2D models even if they use the same iPSC cell line, notably in the absence of an increase in the Aβ_42_/Aβ_40_ ratio, which is one of the best biomarkers observed in the human AD brain. It has been shown that small alterations of Aβ concentrations that affect the Aβ_42_/Aβ_40_ ratio ultimately lead to changes in biophysical and biological properties of the Aβ. Variations in the Aβ pool influence the kinetics of aggregation and the capacity to form amyloid fibrils in vitro and in vivo [19]. Moreover, no 3D model of sporadic AD has been described yet, even though it represents almost 95% of human cases[20].Importantly, iPSC generation involves complete cell reprogramming, which eliminates the phenotypes associated with cellular aging, such as mitochondrial function and telomere length, which are returned to a “embryonic-like” state [21,22]: this means that even if iPSC cell lines originate from sporadic AD patients [13], reprogramming may have erased a variable proportion of the epigenetic marks responsible for a favorable genetic background for the disease, allowing full recapitulation of AD hallmarks.

With all of that in mind, our aim was to change the paradigm by defining a novel induction strategy for a typical AD signature in control iPSCs from healthy people. We aimed to reproduce the Aβ fluctuations observed in AD in vivo and in vitro, which support the amyloid cascade hypothesis [4,23,24], in brain organoids from control iPSCs, to establish a new model of sporadic AD. Physiological maturation of the MBs in vitro was studied through the expression of Aβ amyloids and PrPc, which is the main receptor for Aβ oligomers, during the first months of culture. An Amyloid-β Forty-Two Inducer, a pharmacological compound also known as Aftin-5, was used on MBs to influence APP post-translational pathways and to alter the generation of Aβ forms, producing an imbalance between Aβ_42_ and Aβ_40_ in 2D culture models[25]. The results reported here show for the first time that after chemical induction, MBs can reproduce the phenotype of the increased Aβ_42_/Aβ_40_ ratio, which is observed in both familial and sporadic AD patients.

## Results

### Generation and characterization of mini-brains

**Fig 1:**
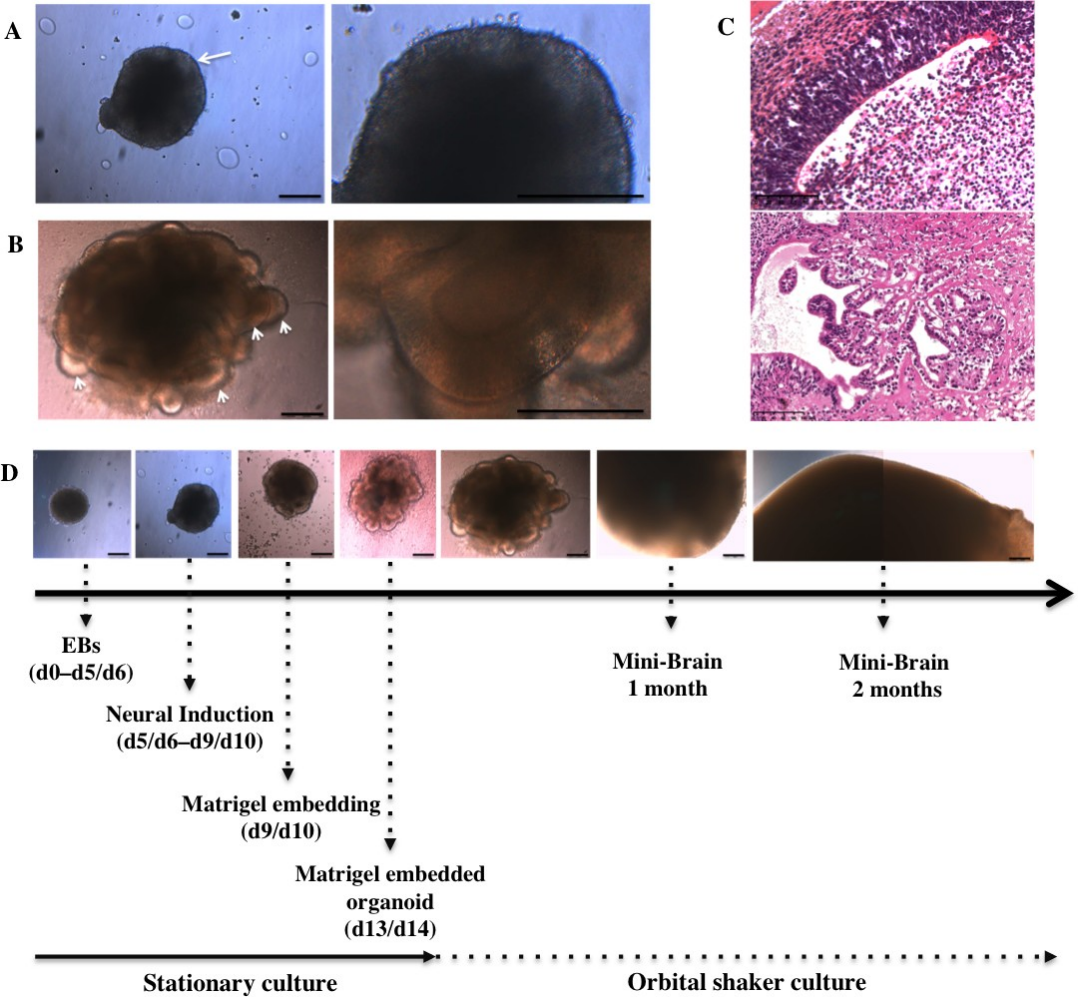
Mini-Brain maturation through neural induction and differentiation. (A) Neural induction matures the neuroectodermic layers at the outer surface of the EB, the edge of which appears optically translucent (arrow). (B) Neural differentiation in Matrigel droplets expands neuroepithelial buds (arrowheads) and increases their size (primitive organoid). (C) Organoids develop in size and morphology for 2 months, reaching their maximal size of 3–4 mm in diameter at the end of this period. (D) Hematoxylin and eosin staining shows the presence of discrete brain regions such as ventricle like-cavities (upper panel, 1-month old MBs) and choroid plexus-like structures (lower panel, 4-month old MBs). Scale bars 250 μm (A, B, C) and 100 μm (D).

The MB protocol previously described by Lancaster et al. [26,27] was reproduced with minor modifications (see Methods) from human iPSCs (S1 Fig) to achieve discrete brain regionalization in vitro as confirmed after analysis of the expression of several neural markers. We observed 3D neuroectodermal differentiation towards neuroepithelium and self-organization leading to growth and development of specific brain regions representative of whole brain tissues. Neural ectoderm acquired apicobasal polarity and developed as a continuous layer during neural induction (NI) along the outer surface of EBs (Fig 1A). Neural differentiation and encapsulation of 3D free-floating cell aggregates within the extracellular matrix provided by Matrigel enabled neuroepithelia to organize radially (Fig 1B, higher magnification image on the right). Moreover, the use of an external scaffold that provided a structural support allowed the neuroepithelial buds to expand (Fig 1B, arrowhead) along with further growth, regionalization and differentiation of neuroepithelial tissue (Fig 1C). Finally the MB protocol supported the growth of neural tissue and its continuous expansion, achieving maximal size after 2 months (Fig 1C) as confirmed by previous studies [27,28] as well as the development of 3D neural microenvironments, which allowed the identification of discrete brain regions (Fig 1D).

Immunohistochemical analyses were used to characterize the presence of different neural cell types. Ventricle-like cavities reminiscent of brain ventricles (Fig 1D) expressing specific markers such as Nestin, PAX6, Ki67 and SOX2 suggest the proliferation of neural stem cells (NSCs) (S2A Fig) and real-time PCR analysis confirms increased marker expression associated with regionalization and neural progenitors, along with a decrease in other germ layer markers (S2B Fig). The presence of more mature neurons was shown by the expression of neuronal proteins such as NeuN, NCAM, MAP2, TUJ1, and CTIP2 indicating differentiation, migration and maturation of newly formed neurons (S2C Fig). Moreover, staining for intermediate filament proteins GFAP and Vimentin shows the existence of glial cell types (S2D Fig).

### Chemical induction of Aβ accumulation

**Fig 2:**
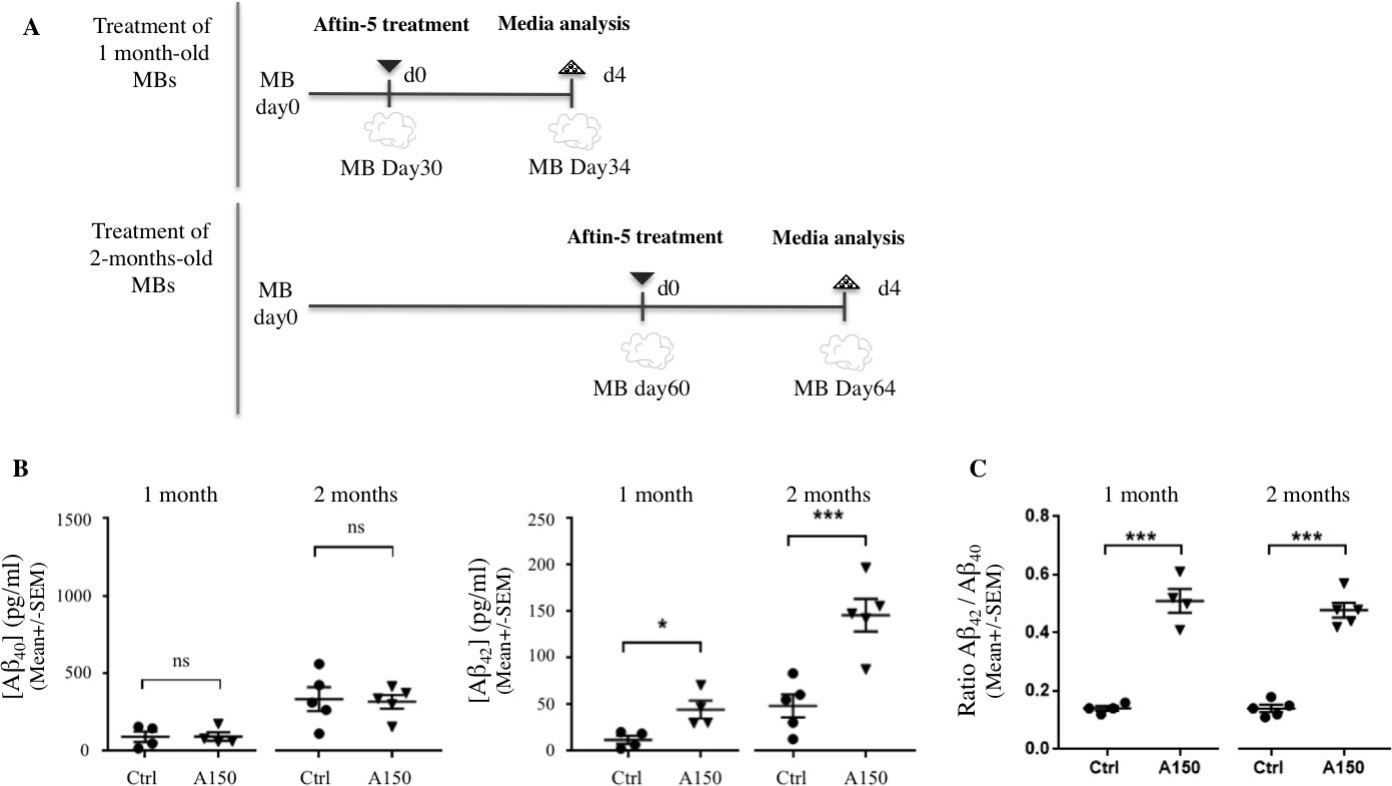
Chemical induction of APP cleavage in vitro upregulates both Aβ_42_ and Aβ_42_/Aβ_40_ ratio without altering Aβ_40_ levels. (A) Schematic representation of treatments with Aftin-5; 1- and 2-month-old MBs (upper and lower representations respectively) were treated once with Aftin-5 for 4 days before collecting conditioned media. (B) Aβ_40_ (left) and Aβ_42_ (right) mean concentration levels in conditioned media measured by ELISA (MSD); concentrations of soluble Aβ_40_ and Aβ_42_ peptides from supernatant of vehicle-treated with DMSO (Ctrl) were compared with concentrations obtained from MBs treated with Aftin-5 (concentration of 150μM (A150)). (C) Aβ_42_/Aβ_40_ ratios corresponding to 1 month- and 2-month old organoids measured by ELISA (MSD) in conditioned media of MBs treated with control vehicle or with Aftin-5. Statistical analysis: unpaired nonparametric Mann-Whitney test. On charts *: p = 0.02; ***: p = 0.009; ns: not significant.

MBs develop discrete brain regions after one month of culture [28] and reach their maximal size of 3–4 mm in diameter after 2 months (Fig 1. and [27]). To investigate whether MBs can be used to present an increased Aβ_42_/Aβ_40_ ratio, MBs were treated with Aftin-5, (Amyloid-β Forty-Two Inducer) a chemical compound which increases the production of extracellular soluble secreted amyloid peptides in vitro [25]. We measured Aβ_42_ and Aβ_40_ concentrations in conditioned media after Aftin-5 induction in 1-month old and 2-month old MBs (Fig 2A).

Chemical treatment of MBs was found to alter APP catabolism, leading to changes in extracellular Aβ_42_ production without alteration of Aβ_40_ concentration (Fig 2B). Statistical data analysis showed significantly higher Aβ_42_ mean levels in 1 month- (*p = 0.02) and 2-month old (***p = 0.009) MBs following treatment with Aftin-5 when compared to vehicle-treated control MBs. An increase in the Aβ_42_/Aβ_40_ ratio was observed consequently (Fig 2C), consistent with previously published in vitro and in vivo 2D models. Interestingly, higher Aβ_42_ production was observed after Aftin-5 treatment of 2-month old MBs compared to 1-month old MBs. This could be a consequence of the maturation of neuroectodermal organoids occurring between 1 and 2 months. Moreover, after treatment with aftin, organoids displayed no observable phosphorylation of tau protein, nor extracellular β-amyloid as shown by immunohistochemical staining (S4 Fig).

### Time-dependent physiological expression of Aβ oligomers during culture

To establish the physiological expression of Aβ during MB culture, Aβ levels were compared at different timepoints. The concentration of soluble Aβ peptides measured in conditioned media harvested from 1-month old MBs was compared to that harvested from 2-month old MBs. During physiological maturation of MBs, Aβ levels increased with age (S3 Fig): A significant increase in concentrations of secreted Aβ fragments was observed in 2-month-old MBs when compared to 1-month-old cultures. Both Aβ_40_ and Aβ_42_ concentrations increased during MB maturation without alteration in Aβ_42_/Aβ_40_ ratios (S3 Fig). These data suggest that during MB maturation there is an accumulation of Aβ peptides, while the ratio remains stable, which is consistent with a physiological condition.

### APP and PrP^C^ expression analysis throughout maturation of MB culture

**Fig 3:**
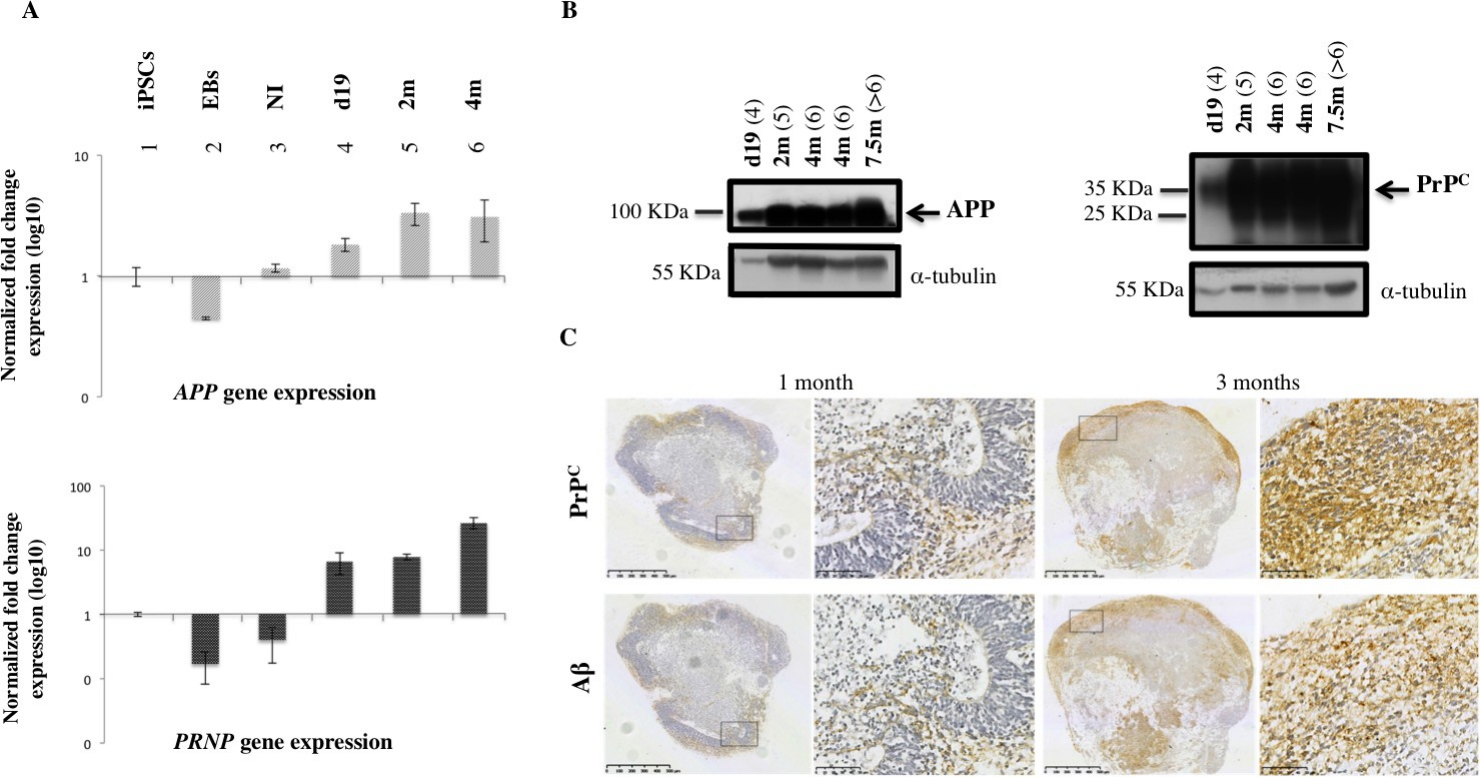
APP and PrP^C^ expression in MBs during culture. (A) Real-time PCR analysis shows *APP* and *PRNP* gene expression at different times during culture. Expression during MB generation and differentiation was normalized with iPSCs expression, which was used as the baseline. (1) iPSCs; (2) embryoid bodies, (EBs); (3) embryoid bodies after neural induction (NI); (4) MBs after 19 days of culture (d19); (5) 2-month old MBs (2m); (6) 4-month old MBs (4m). (B) Western Blot analysis of APP and PrP^C^ using specific antibodies: PrP^C^ antibody SAF32 is specific for the octapeptide repeat region at the N-terminal site which contains the octarepeat region; full-length diglycosylated PrPC appears at ∼ 35 kDa (arrow) whereas unglycosylated and truncated forms appear at a lower molecular weight. 22C11 antibody recognizes amino acid sequence 66–81 and allows identification of a full-length APP695 isoform at ∼ 100 kDa (arrow) as well as an N-truncated form of APP protein. The Bradford method was used to measure the protein load (5 μg of total protein per well); α-tubulin (∼ 55 kDa) was used as loading control. (4) MBs after 19 days of culture (d19); (5) 2-month old MBs (2m); (6) 4-month old MBs (4m); (>6) 7-month old MBs (7m). (C) Tissue sections of 1 month- (left panel) and 3-month old MBs (right panel), immunostained with specific antibodies recognizing PrP^C^ (SAF32 antibody, specific for an N-terminal epitope of protein, upper panel) and Aβ peptides (BAM10 antibody, lower panel).

We have shown that cerebral organoid maturation and cellular type enrichment occur in a time-dependent manner during up to 6 months of culture [28]. We investigated the expression level of relevant genes during MB maturation. Research for a specific Aβ_42_ cellular surface receptor has led to the identification of a highly specific interaction with the cellular prion protein (PrP^C^) [29]. Once the expression pattern of secreted Aβ oligomers was established in the MB model, we chose to study the expression and metabolism of APP and PrP^C^ throughout the maturation of MB (Fig 3A and 3B). Transcript and protein expression were analyzed during 7.5 months of culture (Fig 3A and 3B). The expression levels of APP and PrP^C^ were modulated in a time-dependent manner during culture. *APP* and *PRNP* gene expression increased after neural induction (NI) (Fig 3A) and protein analysis confirmed time-dependent production of APP and PrP^C^ (Fig 3B). Finally, immunohistochemistry of MBs was used to localize PrP^C^ and Aβ expression and to compare 1-month and 2-month old MBs. Immunohistochemical analysis confirmed the existence of increased immunoreactivity between 1 and 3 months using an N-terminal specific antibody for PrP^C^ (SAF32) and an antibody directed against the N-terminal part of Aβ peptide sequence (clone BAM10) (Fig 3C). Aβ and PrPc were expressed in the same regions of the organoid (Fig 3C), suggesting a coexpression of the two biomarkers. These data correlate with the parallel expression signatures of these two genes during the early phases of brain development (see human transcriptome available online: http://hbatlas.org). These data confirm that time-dependent changes in APP metabolism, which correspond to increased Aβ production found in conditioned media (S3 Fig) as well as in MB sections (Fig 3C), correlate with the increase in PrP^C^ expression, confirming a potential role for these proteins during cerebral tissue maturation in vitro.

## Discussion

Human MBs have been shown to be a highly valuable and predictive in vitro tool to study human brain physiological pathways during development as well as a powerful means of recapitulating brain-associated pathologies [17,18,27,30]. The MB model is considered to be very promising in the future study of neurological and neurodegenerative disorders [31]. One remaining question is whether human neural cells issued from iPSCs provide an adequate model for recapitulating phenotypes associated with neurodegenerative diseases. Because reprogramming to iPSC reverts cells to a fetal state, this could represent a problem in modelling age-related neurological disorders. However, several studies have demonstrated and validated the use of iPSCs to model pathologies such as AD. Cultures of patient-derived neurons have shed light on AD pathogenic mechanisms confirming the utility and the substantial potential of patient-derived iPSCs obtained from sporadic or familial forms of the disease [32]. These models show high variability, where a single cell line can give rise to different results depending on the differentiation protocol. For instance, the same FAD cell line may demonstrate contrasting results when used in 2D neuronal assays compared 3D brain organoid assays (S1 Table). Critical improvements are needed to obtain more reproducible models of AD pathology based on these systems, especially concerning the sporadic form of AD, which represents most clinical cases.

We followed the secretion of Aβ oligomers by MBs obtained from a control IPS cell line to evaluate the possibility of provoking an AD-associated phenotype through chemical induction. It has been shown that increased production of the longer Aβ species (such as Aβ_42_ or Aβ_43_) makes aggregation more likely, and leads to the formation of toxic oligomers which confers synaptic dysfunction and ultimately may give rise to amyloid plaque formation [33]. A shorter and more hydrophilic Aβ_40_ form, which is the most abundantly produced Aβ fragment, is thought to contribute directly to small changes observed in Aβ pool concentrations [4,34,35]. The Aβ_42_/Aβ_40_ ratio has been shown in many preclinical and clinical models to be more biologically relevant for AD than Aβ_42_ level alone [33,36,37].

In this work, a normal control IPS cell line differentiated in neuroectodermal organoids displayed a physiological time-dependent increase in Aβ secretion and a stable Aβ_42_/Aβ_40_ ratio over the first two months of culture, reminiscent of the natural maturation occurring in humans. This suggests that although IPS-derived organoids remain embryonic in nature, maturation occurs in vitro. A chemical induction strategy was adopted to modulate APP cleavage and to increase the susceptibility of MBs to Aβ accumulation mechanisms. Induction with Aftin-5 reproducibly led to disruption in Aβ_42_/Aβ_40_ ratios due to a rapid increase of soluble Aβ_42_ accumulation after 4 days of treatment, thus acquiring the specific AD signature. Our data shows that the MB model can be used for the specific purpose of studying AD phenotypes, on a normal genetic background, reminiscent of the signature of sporadic AD.

Some accounts evaluate that sporadic AD affects up to 50% of the population by the age of 85 [20]. This evidence suggests that the basic genetic component of sporadic AD is not the main etiological factor in most of these cases and orientates to alternative hypotheses as non-sequence-based sources of variability in the genome, notably epigenetic modifications (which encompass the different conformational changes in DNA and/or chromatin). It has been reported that >30% of chromatin is dramatically reorganized in senescent cells [38]. Moreover, multiple aging models strongly suggest a massive alteration in histone modification patterns, while multiple genes are directly affected by an altered DNA methylation (global DNA hypomethylation and local hypermethylation that may activate specific transcriptional programs). If old cells accumulate aggregated proteins, genomic damage and display telomere erosion and mitochondrial dysfunction, their reprogramming into iPSC can be considered to mimic the “resetting” that occurs during meiosis and fertilization, which allows the formation of a new individual from two older individuals. [21]. In other words, the most critical epigenetic modifications expected from the cells from AD patients are potentially neutralized during the reprogramming process to generate iPSC cell lines. Moreover, depending on the differentiation protocols used to obtain 2D or 3D neuronal or more complex models such as MBs, different epigenetic marks will be created, i.e. a different impact on the underlying genetic component.

The consequence of Aβ peptide production, accumulation and interaction with other proteins is still under study, but prion-like mechanisms are known to underlie most human neurodegenerative disorders including AD and Parkinson’s disease [39]. Because PrPc acts as a high-affinity receptor for Aβ oligomers [29] and this interaction produces a neurotoxic effect in mice [29], we investigated if PrPc was expressed during the first stages of development of the organoid. We found that the expression of APP and PrPc are both increased during the first two months of culture.

It has been suggested that PrPc extracted from AD brains contains multiple specific binding sites depending on the C-terminal of the Aβ fragment as well as its biophysical conformation at multiple sites on the protein [40,41], but the consequences of the strong interaction between PrPc and Aβ species remain to be established in the AD context. Moreover very little is known about the physiological roles of these proteins in brain development and physiology [42–45]. This study has shown for the first time a spatial and temporal correlation between PrPc and APP/Aβ expression during normal MB maturation, which suggests physiological interactions and opens up new avenues for further investigations.

Confronted with current limits linked to the use of transgenic mouse models that reflect neither the complexity of the human brain nor all the different aspects of AD pathology, MBs constitute a unique human brain model describing time-dependent expression and metabolism of APP and PrP^C^ during long-term culture as well as a model in which Aβ_42_ amyloid secretion can be chemically induced.

## Conclusion

To surmount prevailing obstacles inhibiting the development of efficient strategies in the treatment of AD, the pharmaceutical industry requires alternative experimental models. Although progress has been made using transgenic mouse models, to date no in vivo model reflects the complexity of the human brain or reproduces the full extent of the pathology found in patients. Comparing the phenotypes obtained using various 2D in vitro cell culture methods highlights discrepancies between the models as well as their inability to coherently recapitulate pathological events. 3D-based models may constitute a more physiologically relevant model, although the embryonic nature of IPS derivatives may hinder this type of approach. The aim of this work was to take advantage of the research prospects offered by neuroectodermal organoids in the field of neuroscience. To validate the use of MB models to recapitulate AD-associated phenotypes, the ability of the chemical inductor Aftin-5 to modulate the APP post-transcriptional pathway towards a pathological outcome was tested on organoids obtained from a control IPS cell line without any described mutations. MBs were chosen for this study to investigate the proteins implicated in pathophysiological mechanisms linked to sporadic forms of AD. Treatment of MBs with Aftin-5 leads to a reproducible disruption of the physiological balance of the Aβ_42_/Aβ_40_ ratio observed during normal maturation. The secreted Aβ_42_ concentration and Aβ_42_/Aβ_40_ ratio rose over 4 days of treatment whereas the secreted Aβ_40_ concentration remained stable. In this timeframe, neither phosphorylation of tau protein nor extracellular β-amyloid were revealed by immunohistochemistry in the aftin-treated organoids (S4 Fig). Our study supports the pioneering work of Raja et al., who aimed to closer emulate human pathology using a 3D in vitro model of the brain based on FAD iPSC to study neurological disease, although further work is still needed in order to recapitulate all the hallmarks of AD in vitro.

Our aim was to test the capacity of the MBs to model sporadic AD even when the initial iPSC cell line originated from a non-AD patient. We used a strategy of chemical induction to reproduce the fluctuations in Aβ observed in AD which support the amyloid cascade hypothesis in brain organoids issued from control iPSCs [4,23,24]. We confirmed that human MBs obtained from a normal patient can reproduce a relevant phenotype in vitro, opening new perspectives in the understanding of physiological and pathological brain mechanisms. We further demonstrate the possibility of studying the expression of biomarkers during neural differentiation and maturation over several months in vitro, validating a physiological expression profile for APP and PrP^C^ over time. This is consistent with human brain development and maturation in vivo. In addition, we were able to identify a physiological Aβ accumulation in vitro which differs from that obtained using Aftin-5. In particular, Aβ_40_ and Aβ_42_ levels were found to increase with time while maintaining a stable ratio during the first two months of culture.

Through the observation of APP metabolism and PrP^C^ expression, we show that after chemical induction, the MB model of 3D maturing human cells, capable of long-term culture, can be used to study phenotypes associated with sporadic cases of neurodegenerative pathologies. This concept can also be tested in AD derived IPSCs to compensate for critical phenotypical signatures that could have been lost during reprogramming. Future improvements to this model such as the integration of vascularization and the combination of genetic and molecular strategies to accelerate normal aging processes may contribute to the identification of novel biomarkers or potential targets in AD. Chemical induction of AD-related proteins in neuroectodermal organoids provides a biochemical signature that is closer to the most frequent sporadic forms of AD, which could provide a path towards more predictive future models applicable in the field of drug discovery.

## Experimental procedure

### Maintenance of iPSCs culture and 3D differentiation of mini-brains

BJ primary fibroblasts were obtained from ATCC (CRL-2522). Fibroblasts were reprogrammed using the Sendai virus reprogramming method as recommended by the manufacturer (Life Technologies, cat. no. A16517). Briefly, BJ cells were infected on day 0, and plated on inactivated mouse embryonic fibroblasts (MEF, GlobalStem) 48 hours after infection. Stem cell medium composed of DMEM/F12 (Life Technologies), supplemented with 20% KOSR (Life Technologies), glutamine, non-essential amino-acids and bFGF (10 ng/ml, Stem Cell Technologies,) was changed daily. Clones were picked starting 20 days post infection, and expanded on murine embryonic fibroblasts (MEF, GlobalStem) before being adapted to feeder-free conditions. Assessment of pluripotency by semi-quantitative PCR was performed periodically to confirm the expression of pluripotent markers in iPSC cultures (S1 Fig). iPSCs used to obtain cerebral organoids were maintained in feeder-free condition on Matrigel-coating (Corning), fed using mTeSR1 medium (StemCell Technologies) and checked to confirm the absence of mycoplasma. iPSC colonies were cultured up to 80% of confluence. We followed the protocol published by Lancaster et al. with minor modifications [26]. At day 0 of the differentiation protocol cells were dissociated to allow cell counting as described previously. The first step of the previously published protocol was modified using a more homogeneous method to obtain embryoid bodies (EBs) [46]. Briefly, hanging drops of 20 μl of EBs medium containing 9000 cells per drop were cultured on a Petri dish cover (10 cm in diameter) for 2 days in order to allow them to aggregate before being collected (S5A Fig). At day 2 each EB was harvested manually using a cut micropipette tip and placed in a well of a non-treated 24-well plate (S5B Fig) (Corning). Media changes, neural induction and neural differentiation were performed following the published protocol [26].

### Immunohistochemical analysis

MBs were washed twice with calcium and magnesium-free D-PBS (Sigma-Aldrich) and fixed in formaldehyde (4%) (VWR) solution for 15 minutes at 4°C and then 72 hours at RT. MBs were washed again in D-PBS and prepared for paraffin embedding (Leica TP 1050). Histological paraffin sections of 5 μm were prepared using a microtome (Leica) and collected on glass microscope slides (Thermo scientific). For immunostaining, paraffin was removed in a Medite-Medixintechnik Tissue Stainer TST 40 device and pretreated for epitope retrieval. We performed HIER (heat-induced epitope retrieval) pretreatment combining a heat treatment at 95°C with a citrate buffer solution (pH 6.0) for 40 minutes. Slides were then recovered, washed in MilliQ warm water, blocked, washed twice with MilliQ water, once with PBS solution 1X (Sigma-Aldrich) and once with TBS-Tween 0.05% solution (Sigma-Aldrich). Sections were then incubated with 200 μl of primary antibody solution overnight at 4°C. The antibody solution was removed and sections received three washes of TBS-Tween 0.05% solution (Sigma-Aldrich) with agitation using an orbital shaker before the successive incubation with the secondary antibody (ImmPress Reagent Kit peroxidase) for 30 minutes at RT. TBS-Tween 0,05% washes were repeated and 200 μl of DAB reagent per section (ImmPACT Peroxidase substrate kit) was incubated (for 5 to 10 minutes) before being subjected to nuclear staining procedure in hematoxylin solution (600 μl per section for 5 minutes). Slides were processed automatically (Medite-Medixintechnik Tissue Stainer) to allow tissue dehydration. Finally sections were coverslipped using Eukitt (Sigma-Aldrich) mounting medium and allowed to dry overnight at RT. The primary antibodies used for MB characterization as well as for the study of APP and PrP^C^ expression were diluted as follows: anti-Nestin antibody (Abcam), 1:500; anti-PAX6 (Covance), 1:300; anti-Ki67 (Dako), 1:200; anti-SOX2 (Epitomics), 1:300; anti-NeuN (Chemicon), 1:1000; anti-NCAM (Santa Cruz), 1:100; anti-MAP2 (Sigma), 1:250; anti-TUJ1 (Covance), 1:5000; anti-CTIP2 (Abcam), 1:100; anti-GFAP (Dako), 1:4000; anti-Vimentin (Dako), 1:2000; antibody directed against N-terminal residues of APP 66–81 (22C11, Millipore), 1:100; antibody directed against residues 1–12 of Aβ peptide (BAM10, Sigma-Aldrich), 1:1000; antibody directed against octapeptide region of PrP^C^ (SAF32, provided by J. Grassi, Commissariat á l’Energie Atomique/Saclay, Gif sur Yvette, France), 1:1000.

### Analysis of gene expression

Total RNA was extracted from iPSCs and organoids using a Nucleospin RNA II Kit (Macherey-Nagel) according to the manufacturer's instructions. Reverse transcription (RT) was carried out using an Iscript cDNA synthesis kit (Biorad) following the instructions provided by the manufacturer. A total of 1 μg of RNA was used in RT reactions. PCR amplifications were performed using the Real-Time PCR Mini-Opticon System (Biorad, USA) and the IQSYBR Green Supermix kit (Biorad, USA) according to the manufacturer's protocol. Two microlitres of the first strand cDNA (1:5 diluted) were used for amplification in triplicate in a 10 μL reaction containing SYBR Green and 10 pmol of each primer. The following PCR program was used: 95°C for 3 min, 40 amplification cycles at 95°C for 10 s, 60°C for 30 s. Serial dilutions of each studied transcript were used to determine the amplification efficiency of each target and housekeeping gene (TBP or RPLP0). Data were obtained by analysis with BioRad MFX Software 2.0 and are presented as the fold-change in target gene expression normalized to the internal control gene. The average threshold cycle (CT) was calculated for both the target gene and housekeeping genes, and ΔCT was determined as [the mean of the triplicate CT values for the target gene]-[the mean of the triplicate CT values for TBP or RPLP0]. ΔΔCT represented the difference between the paired samples, as calculated by the formula ΔΔCT = (ΔCT of sample—ΔCT of control).

### Aβ quantification

The concentration of soluble Aβ_42_, Aβ_40_ and Aβ_38_ fragments produced after Aftin-5 treatment was measured by Enzyme-linked immunoabsorbent assay (ELISA) using reagents, protocol and imager manufactured by Meso Scale Diagnostic (MSD Multi-Spot Assay System) and compared to the control (DMSO). After four days of treatment the medium was collected and a multiplex kit containing Aβ peptide panel (4G8 and 6E10 Aβ antibodies) was applied according to the manufacturer’s protocol.

### Statistical analysis

Data were analyzed using PRISM 7 (GraphPad) and SPSS (IBM Analytics) analysis software and values are expressed as the means ± SD. Means of concentrations and ratios were calculated from independent experiments and values corresponding to each mini-brain were represented by a circle (controls) or a triangle (treated). Each point represents a conditioned medium obtained from the culture of a single organoid. Statistical significance was tested using a nonparametric Mann-Whitney test to compare the differences between groups. Differences were retained as statistically significant using p-values of < 0.05 (confidence level 95%).

## Supporting information

S1 Fig Assessment of pluripotency. Assessment of pluripotency by semi-quantitative PCR to confirm the expression of pluripotent markers by iPSC line BJ. PCR analysis shows the expression of pluripotency markers *OCT4*, *SOX2*, *NANOG* and *REX-1*. BJ fibroblasts were used as a control and *RPLP0* as a housekeeping gene.
(TIF)

S2 Fig **Characterization of MBs** (A) Immunohistochemical (IHC) staining of neurogenic niches shows the expression of markers associated with neural stem cell maintenance (Nestin, PAX6, SOX2) and proliferation (Ki67) at the apical surface of ventricular-like cavities. (B) Quantitative PCR analysis of neural markers which compare undifferentiated iPSCs with differentiated EBs and MBs (after 19 days of differentiation); increased genetic expression of ectodermal (NES and OLIG2), associated markers accompanied by a decrease of endoderm (AFP) and mesoderm (BRACH) markers after 19 days culture.
OLIG2 expression indicates also ventral and dorsal regionalization of the spinal cord as well as the presence of oligodendrocytic precursor identities. The expression of forebrain (FOXG1) and hindbrain (ISL-1) markers confirms MB regionalization during differentiation. (C) IHC staining allows the identification of neural marker expression which indicates the presence of more mature neurons suggesting the differentiation and migration of NSCs from a neurogenic niche. NeuN, neuron specific nuclear protein; NCAM, neural cell adhesion molecule; MAP2, microtubule associated protein 2; TUJ1, neuron specific class III β-tubulin; CTIP2, newly born deep layer neurons. (D) Glia identity was established using intermediate filament specific antibodies GFAP (glial fibrillary acidic protein) and Vimentin.
(TIF)

S3 Fig Analysis of Aβ_40_, Aβ_42_ and Aβ_42_/Aβ_40_ ratio in conditioned media after 1 and 2 months of MB culture; Statistical analysis: unpaired nonparametric Mann-Whitney test. On charts *: p **=** 0.002; ***: p **=** 0.009; ns: not significant.
(TIF)

S4 Fig Immunohistochemical staining of Aftin 5-treated MB. IHC with 12F4 (anti-Aß42) and AT8 (anti-phospho-Tau(Ser202,Thr205). A- Staining controls: IHC stained sections with 12F4 antibody of postmortem brain samples: on the left non-human primate, adult sample (1), on the right human late stage AD sample (2) B- Staining of organoids: IHC stained sections with 12F4 antibody of Minibrains: on the left MB treated with vehicle (DMSO) (1), on the right MB treated with Aftin-5 (2) C- Staining controls: IHC stained sections with AT8 antibody of postmortem brain samples: on the left healthy non-human primate adult sample (1), on the right human late-stage AD sample (2) D- IHC stained sections with AT8 of Minibrains: on the left MB treated with vehicle (DMSO) (1), on the right MB treated with Aftin-5 (2). Scale bars: 100 μm.
(TIF)

S5 Fig Generation of MBs using hanging drop method. (A) iPSC colony cultured on Matrigel before dissociation (corresponding to day 0 of the MB protocol, on the left), hanging drop culture of iPS cell suspension on a petri dish cover (day 0—day 2, second picture); iPSCs were maintained in drop culture for 2 days allowing cells to aggregate, forming the EB in the center of the hanging drop and each drop contains one EB (third picture) which is harvested at day 2 (right). (B) Representation of the two step hanging drop method; EBs were cultured in the hanging drops for 2 days; then, each EB was recovered manually with a cut micropipette tip and placed into a 24-well plate containing EB medium.
(TIF)

S1 Table Comparison of iPSC lines used to reproduce AD-associated phenotypes in 2D and 3D neural cultures *in vitro*. In the study of Raja et al. 2016, all four FAD lines were used to create 3D brain organoids. APPDp 1–1, APPDp 2–3 (EOAD) carrying APP duplication were used in the study of Israel et al. 2012 whereas FAD lines ND34732 (PSEN1 M146I) and AG06840 (PSEN1 A264E) were used in other studies as indicated in the respective lines. Results indicated in the table refer to extracellular measures of Aβ peptide concentrations (Duan et al. 2014; Yagi et al. 2014; Liu et al. 2014; Sproul et al. 2014).(□) Increase in the concentration of Aβ compared to control lines; (=) no change in the concentration observed between FAD and control lines; (/) unknown.
(TIF)

## References

[1] Kitazawa M, Medeiros R M. LaFerla F. Transgenic Mouse Models of Alzheimer Disease: Developing a Better Model as a Tool for Therapeutic Interventions. Curr Pharm Des. 2012;18: 1131–1147. 10.2174/138161212799315786 22288400 10.2174/138161212799315786 PMC4437619

[2] Cummings J. Lessons Learned from Alzheimer Disease: Clinical Trials with Negative Outcomes. Clin Transl Sci. 2018;11: 147–152. 10.1111/cts.12491 28767185 10.1111/cts.12491 PMC5866992

[3] Lesné S, Ming TK, Kotilinek L, Kayed R, Glabe CG, Yang A, et al A specific amyloid-β protein assembly in the brain impairs memory. Nature. 2006;440: 352–357. 10.1038/nature04533 16541076 10.1038/nature04533

[4] Haass C, Selkoe DJ. Soluble protein oligomers in neurodegeneration: lessons from the Alzheimer’s amyloid β -peptide. Nat Rev Mol Cell Biol. 2007;8: 101–112. 10.1038/nrm2101 17245412 10.1038/nrm2101

[5] Cavanaugh SE. Animal models of Alzheimer disease: historical pitfalls and a path forward. ALTEX. 2014;31: 279–302. doi: 10.14573/altex.1310071 24793844 10.14573/altex.1310071

[6] De Strooper B. Lessons from a failed γ-secretase Alzheimer trial. Cell. Elsevier Inc.; 2014;159: 721–726. 10.1016/j.cell.2014.10.016 25417150 10.1016/j.cell.2014.10.016

[7] Goldstein LSB, Reyna S, Woodruff G. Probing the secrets of Alzheimer’s disease using human-induced pluripotent stem cell technology. Neurotherapeutics. 2015; 10.1007/s13311-014-0326-6 25534395 10.1007/s13311-014-0326-6 PMC4322074

[8] Freude K, Pires C, Hyttel P, Hall V. Induced Pluripotent Stem Cells Derived from Alzheimer’s Disease Patients: The Promise, the Hope and the Path Ahead. J Clin Med. 2014;3: 1402–1436. 10.3390/jcm3041402 26237610 10.3390/jcm3041402 PMC4470192

[9] Parr CJC, Yamanaka S, Saito H. An update on stem cell biology and engineering for brain development. Mol Psychiatry. 2017;22: 808–819. 10.1038/mp.2017.66 28373686 10.1038/mp.2017.66

[10] Muratore CR, Srikanth P, Callahan DG, Young-Pearse TL. Comparison and optimization of hiPSC forebrain cortical differentiation protocols. PLoS One. 2014;9: 1–18. 10.1371/journal.pone.0105807 25165848 10.1371/journal.pone.0105807 PMC4148335

[11] Israel M a., Yuan SH, Bardy C, Reyna SM, Mu Y, Herrera C, et al Probing sporadic and familial Alzheimer’s disease using induced pluripotent stem cells. Nature. 2012;482: 216–220. 10.1038/nature10821 22278060 10.1038/nature10821 PMC3338985

[12] Kondo T, Asai M, Tsukita K, Kutoku Y, Ohsawa Y, Sunada Y, et al Modeling Alzheimer’s disease with iPSCs reveals stress phenotypes associated with intracellular Aβ and differential drug responsiveness. Cell Stem Cell. Elsevier Inc.; 2013;12: 487–496. 10.1016/j.stem.2013.01.009 23434393 10.1016/j.stem.2013.01.009

[13] Choi SH, Kim YH, Hebisch M, Sliwinski C, Lee S, D’Avanzo C, et al A three-dimensional human neural cell culture model of Alzheimer’s disease. Nature. Nature Publishing Group; 2014;515: 274–278. 10.1038/nature13800 25307057 10.1038/nature13800 PMC4366007

[14] D’Avanzo C, Aronson J, Kim YH, Choi SH, Tanzi RE, Kim DY. Alzheimer’s in 3D culture: Challenges and perspectives. BioEssays. 2015; 10.1002/bies.201500063 26252541 10.1002/bies.201500063 PMC4674791

[15] Fatehullah A, Tan SH, Barker N. Organoids as an in vitro model of human development and disease. Nat Cell Biol. Nature Publishing Group; 2016;18: 246–254. 10.1038/ncb3312 26911908 10.1038/ncb3312

[16] Brennand KJ, Marchetto MC, Benvenisty N, Brüstle O, Ebert A, Izpisua Belmonte JC, et al Creating Patient-Specific Neural Cells for the in Vitro Study of Brain Disorders. Stem Cell Reports. 2015;5: 933–945. 10.1016/j.stemcr.2015.10.011 26610635 10.1016/j.stemcr.2015.10.011 PMC4881284

[17] Gonzalez C, Armijo E, Bravo-alegria J, Mays ABCE, Soto C. Modeling amyloid beta and tau pathology in human cerebral organoids. Mol Psychiatry. Springer US; 2018; 10.1038/s41380-018-0229-8 30171212 10.1038/s41380-018-0229-8 PMC6594704

[18] Raja WK, Mungenast AE, Lin Y-T, Ko T, Abdurrob F, Seo J, et al Self-Organizing 3D Human Neural Tissue Derived from Induced Pluripotent Stem Cells Recapitulate Alzheimer’s Disease Phenotypes. PLoS One. 2016;11: e0161969 10.1371/journal.pone.0161969 27622770 10.1371/journal.pone.0161969 PMC5021368

[19] Kuperstein I, Broersen K, Benilova I, Rozenski J, Jonckheere W, Debulpaep M, et al Neurotoxicity of Alzheimer’s disease Aβ peptides is induced by small changes in the Aβ42 to Aβ40 ratio. EMBO J. 2010;29: 3408–3420. 10.1038/emboj.2010.211 20818335 10.1038/emboj.2010.211 PMC2957213

[20] Hirtz D, Thurman DJ, Gwinn-Hardy K, Mohamed M, Chaudhuri AR, Zalutsky R. How common are the “common” neurologic disorders? Neurology. 2007;68: 326–337. 10.1212/01.wnl.0000252807.38124.a3 17261678 10.1212/01.wnl.0000252807.38124.a3

[21] Mahmoudi S, Brunet A. Aging and reprogramming: A two-way street. Current Opinion in Cell Biology. 2012 10.1016/j.ceb.2012.10.004 23146768 10.1016/j.ceb.2012.10.004 PMC3540161

[22] Sen P, Shah PP, Nativio R, Berger SL. Epigenetic Mechanisms of Longevity and Aging. Cell. 2016;166: 822–839. 10.1016/j.cell.2016.07.050 27518561 10.1016/j.cell.2016.07.050 PMC5821249

[23] Hardy JA, Higgins GA. Alzheimer’s Disease: The Amyloid Cascade Hypotesis. Science (80-). 1992;256: 184–5. 1566067 10.1126/science.1566067

[24] Tanzi RE, Bertram L. Twenty years of the Alzheimer’s disease amyloid hypothesis: A genetic perspective. Cell. 2005;120: 545–555. 10.1016/j.cell.2005.02.008 15734686 10.1016/j.cell.2005.02.008

[25] Hochard A, Oumata N, Bettayeb K, Gloulou O, Fant X, Durieu E, et al Aftins Increase Amyloid-β42, Lower Amyloid-β38, and Do Not Alter Amyloid-β40 Extracellular Production in vitro: Toward a Chemical Model of Alzheimer’s Disease? J Alzheimer’s Dis. 2013;35: 107–120. 10.3233/JAD-121777 23364140 10.3233/JAD-121777 PMC5039020

[26] Lancaster MA, Knoblich JA. Generation of cerebral organoids from human pluripotent stem cells. Nat Protoc. 2014;9: 2329–2340. 10.1038/nprot.2014.158 25188634 10.1038/nprot.2014.158 PMC4160653

[27] Lancaster MA, Renner M, Martin CA, Wenzel D, Bicknell LS, Hurles ME, et al Cerebral organoids model human brain development and microcephaly. Nature. Nature Publishing Group; 2013;501: 373–379. 10.1038/nature12517 23995685 10.1038/nature12517 PMC3817409

[28] Quadrato G, Nguyen T, Macosko EZ, Sherwood JL, Min Yang S, Berger DR, et al Cell diversity and network dynamics in photosensitive human brain organoids. Nature. 2017; 10.1038/nature22047 28445462 10.1038/nature22047 PMC5659341

[29] Laurén J, Gimbel D a, Nygaard HB, Gilbert JW, M S. Cellular Prion Protein Mediates Impairment of Synaptic Plasticity by Amyloid-β Oligomers-Supplementary Information. Nature. 2009;457: 1128–1132. 10.1038/nature07761 19242475 10.1038/nature07761 PMC2748841

[30] Qian X, Nguyen HN, Song MM, Hadiono C, Ogden SC, Hammack C, et al Brain-Region-Specific Organoids Using Mini-bioreactors for Modeling ZIKV Exposure. Cell. 2016;165: 1238–1254. 10.1016/j.cell.2016.04.032 27118425 10.1016/j.cell.2016.04.032 PMC4900885

[31] Brawner AT, Xu R, Liu D, Jiang P. Generating CNS organoids from human induced pluripotent stem cells for modeling neurological disorders. Int J Physiol Pathophysiol Pharmacol. 2017;9: 101–111. 28694921 PMC5498882

[32] Ghaffari LT, Starr A, Nelson AT, Sattler R. Representing Diversity in the Dish: Using Patient-Derived in Vitro Models to Recreate the Heterogeneity of Neurological Disease. Front Neurosci. 2018;12 10.3389/fnins.2018.00056 29479303 10.3389/fnins.2018.00056 PMC5812426

[33] Lewczuk P, Matzen A, Blennow K, Parnetti L, Molinuevo JL, Eusebi P, et al Cerebrospinal Fluid Aβ42/40 Corresponds Better than Aβ42 to Amyloid PET in Alzheimer’s Disease. J Alzheimer’s Dis. 2017;55: 813–822. 10.3233/JAD-160722 27792012 10.3233/JAD-160722 PMC5147502

[34] Fagan AM, Head D, Shah AR, Marcus D, Mintun M, Morris JC, et al Decreased CSF Aβ42 correlates with brain atrophy in cognitively normal elderly. Ann Neurol. 2009;65: 176–183. 10.1002/ana.21559 19260027 10.1002/ana.21559 PMC2763631

[35] Collins-Praino LE, Francis YI, Griffith EY, Wiegman AF, Urbach J, Lawton A, et al Soluble amyloid beta levels are elevated in the white matter of Alzheimer’s patients, independent of cortical plaque severity. Acta Neuropathol Commun. 2014;2: 83 10.1186/s40478-014-0083-0 25129614 10.1186/s40478-014-0083-0 PMC4147157

[36] Lue L, Kuo Y, Roher AE, Brachova L, Shen Y, Sue L, et al Soluble Amyloid β Peptide Concentration as a Predictor of Synaptic Change in Alzheimer’s Disease. Am J Pathol. 1999;155: 853–862. 10.1016/S0002-9440(10)65184-X 10487842 10.1016/s0002-9440(10)65184-x PMC1866907

[37] Spies PE, Slats D, Sjögren JMC, Kremer BPH, Verhey FRJ, Rikkert MGMO, et al The cerebrospinal fluid amyloid beta42/40 ratio in the differentiation of Alzheimer’s disease from non-Alzheimer’s dementia. Curr Alzheimer Res. 2010;7: 470–6. 10.2174/156720510791383796 20043812 10.2174/156720510791383796

[38] Shah PP, Donahue G, Otte GL, Capell BC, Nelson DM, Cao K, et al Lamin B1 depletion in senescent cells triggers large-scale changes in gene expression and the chromatin landscape. Genes Dev. 2013; 10.1101/gad.223834.113 23934658 10.1101/gad.223834.113 PMC3759695

[39] Goedert M. Alzheimer’s and Parkinson’s diseases: The prion concept in relation to assembled Aβ, tau, and α-synuclein. Science (80-). 2015;349: 601–702. 10.1126/science.1255555 26250687 10.1126/science.1255555

[40] Gunther E, Strittmatter S. β-amyloid oligomers and cellular prion protein in Alzheimer’s disease. J Mol Med. 2010;88: 331–338. 10.1007/s00109-009-0568-7 19960174 10.1007/s00109-009-0568-7 PMC2846635

[41] Zou WQ, Xiao X, Yuan J, Puoti G, Fujioka H, Wang X, et al Amyloid-β42 interacts mainly with insoluble prion protein in the Alzheimer brain. J Biol Chem. 2011;286: 15095–15105. 10.1074/jbc.M110.199356 21393248 10.1074/jbc.M110.199356 PMC3083157

[42] Manson J, West JD, Thomson V, McBride P, Kaufman MH, Hope J. The prion protein gene: a role in mouse embryogenesis? Development. 1992;115: 117–22. 1353438 10.1242/dev.115.1.117

[43] Kovacs GG, Zerbi P, Voigtländer T, Strohschneider M, Trabattoni G, Hainfellner JA, et al The prion protein in human neurodegenerative disorders. Neurosci Lett. 2002;329: 269–272. 10.1016/S0304-3940(02)00668-7 12183028 10.1016/s0304-3940(02)00668-7

[44] Adle-Biassette H, Verney C, Peoc’h K, Dauge M-C, Razavi F, Choudat L, et al Immunohistochemical Expression of Prion Protein (PrPC) in the Human Forebrain During Development. J Neuropathol Exp Neurol. 2006;65: 698–706. 10.1097/01.jnen.0000228137.10531.72 16825956 10.1097/01.jnen.0000228137.10531.72

[45] Steele AD, Emsley JG, Ozdinler PH, Lindquist S, Macklis JD. Prion protein (PrPc) positively regulates neural precursor proliferation during developmental and adult mammalian neurogenesis. Proc Natl Acad Sci. 2006;103: 3416–3421. 10.1073/pnas.0511290103 16492732 10.1073/pnas.0511290103 PMC1413927

[46] Sheridan SD, Surampudi V, Rao RR. Analysis of embryoid bodies derived from human induced pluripotent stem cells as a means to assess pluripotency. Stem Cells Int. 2012; 10.1155/2012/738910 22550517 10.1155/2012/738910 PMC3328185

