# Supplementary figures and images for: Small-molecule induction of Aβ-42 peptide production in human cerebral organoids to model Alzheimer's disease associated phenotypes
Source: PLoS One. 2018 Dec 17;13(12):e0209150. doi: 10.1371/journal.pone.0209150 (PMC6296660; doi:10.1371/journal.pone.0209150)

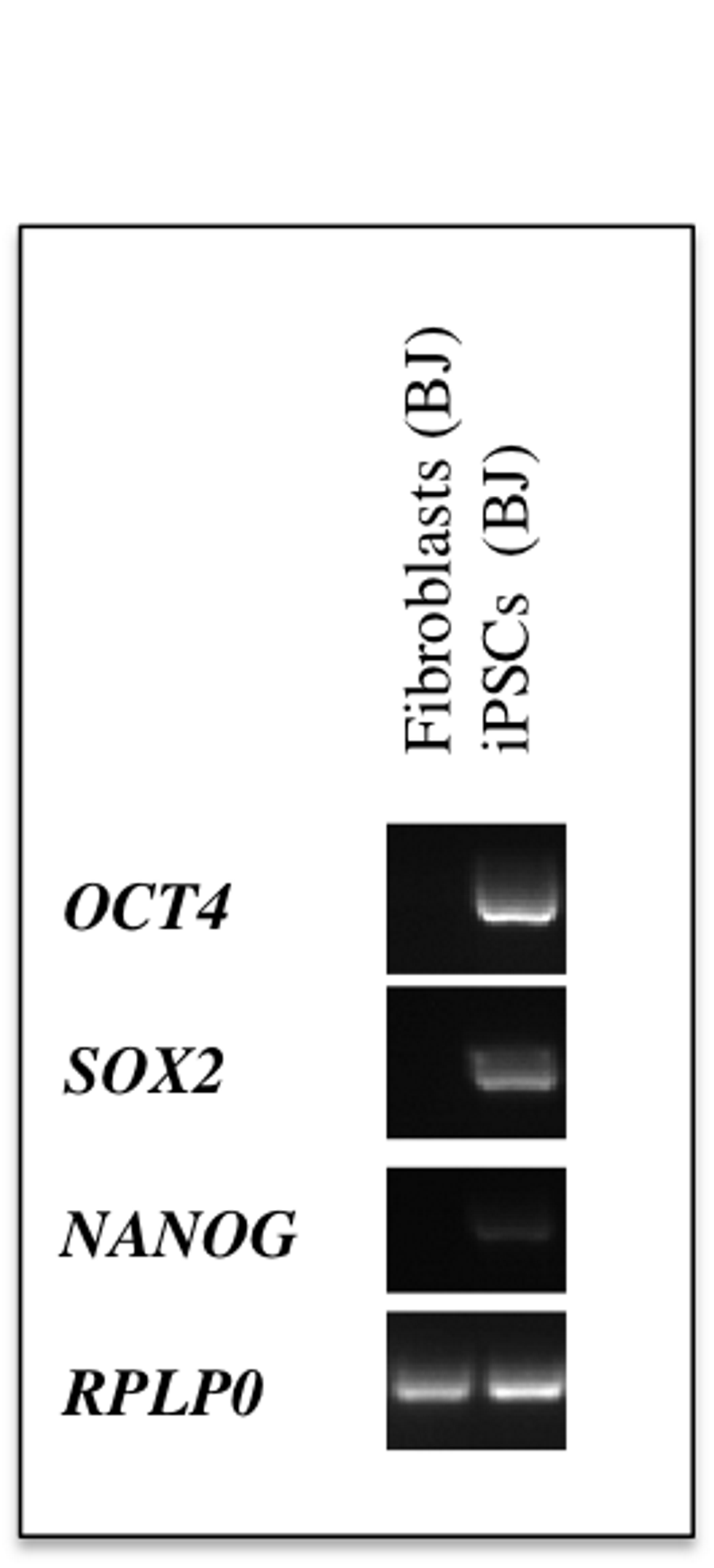

Supplement: S1 Fig — Assessment of pluripotency by semi-quantitative PCR to confirm the expression of pluripotent markers by iPSC line BJ. PCR analysis shows the expression of pluripotency markers OCT4, SOX2, NANOG and REX-1. BJ fibroblasts were used as a control and RPLP0 as a housekeeping gene. (TIF) [file pone.0209150.s001.tif]

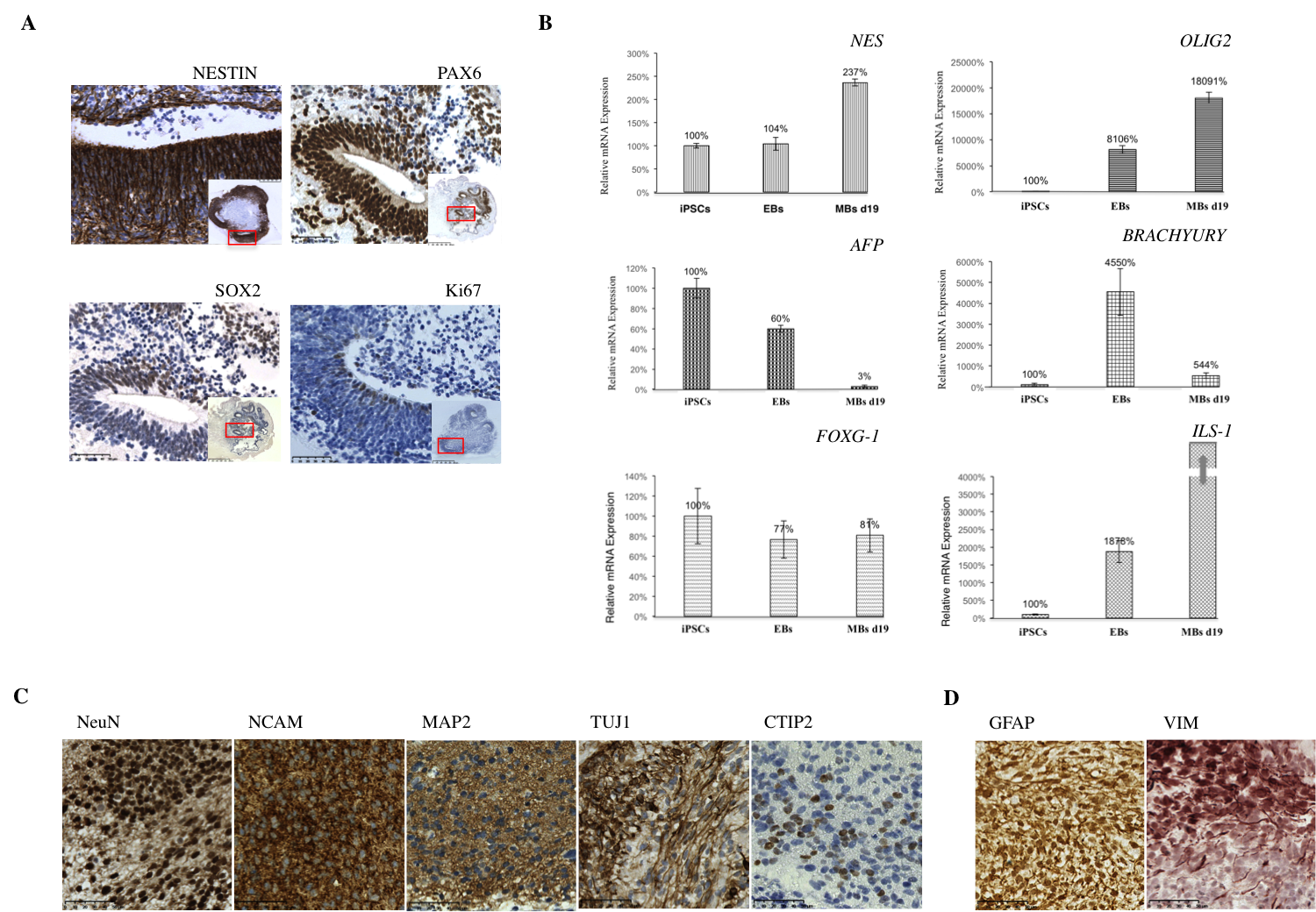

Supplement: S2 Fig — Characterization of MBs (A) Immunohistochemical (IHC) staining of neurogenic niches shows the expression of markers associated with neural stem cell maintenance (Nestin, PAX6, SOX2) and proliferation (Ki67) at the apical surface of ventricular-like cavities. (B) Quantitative PCR analysis of neural markers which compare undifferentiated iPSCs with differentiated EBs and MBs (after 19 days of differentiation); increased genetic expression of ectodermal (NES and OLIG2), associated markers accompanied by a decrease of endoderm (AFP) and mesoderm (BRACH) markers after 19 days culture. OLIG2 expression indicates also ventral and dorsal regionalization of the spinal cord as well as the presence of oligodendrocytic precursor identities. The expression of forebrain (FOXG1) and hindbrain (ISL-1) markers confirms MB regionalization during differentiation. (C) IHC staining allows the identification of neural marker expression which indicates the presence of more mature neurons suggesting the differentiation and migration of NSCs from a neurogenic niche. NeuN, neuron specific nuclear protein; NCAM, neural cell adhesion molecule; MAP2, microtubule associated protein 2; TUJ1, neuron specific class III β-tubulin; CTIP2, newly born deep layer neurons. (D) Glia identity was established using intermediate filament specific antibodies GFAP (glial fibrillary acidic protein) and Vimentin. (TIF) [file pone.0209150.s002.tif]

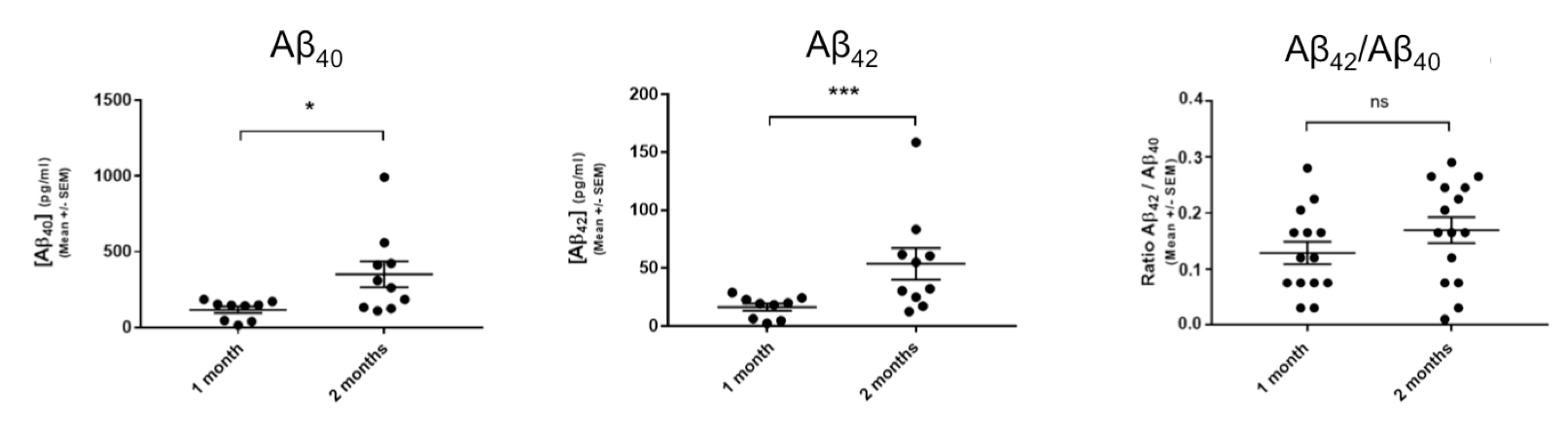

Supplement: S3 Fig — On charts *: p = 0.002; ***: p = 0.009; ns: not significant. (TIF) [file pone.0209150.s003.tif]

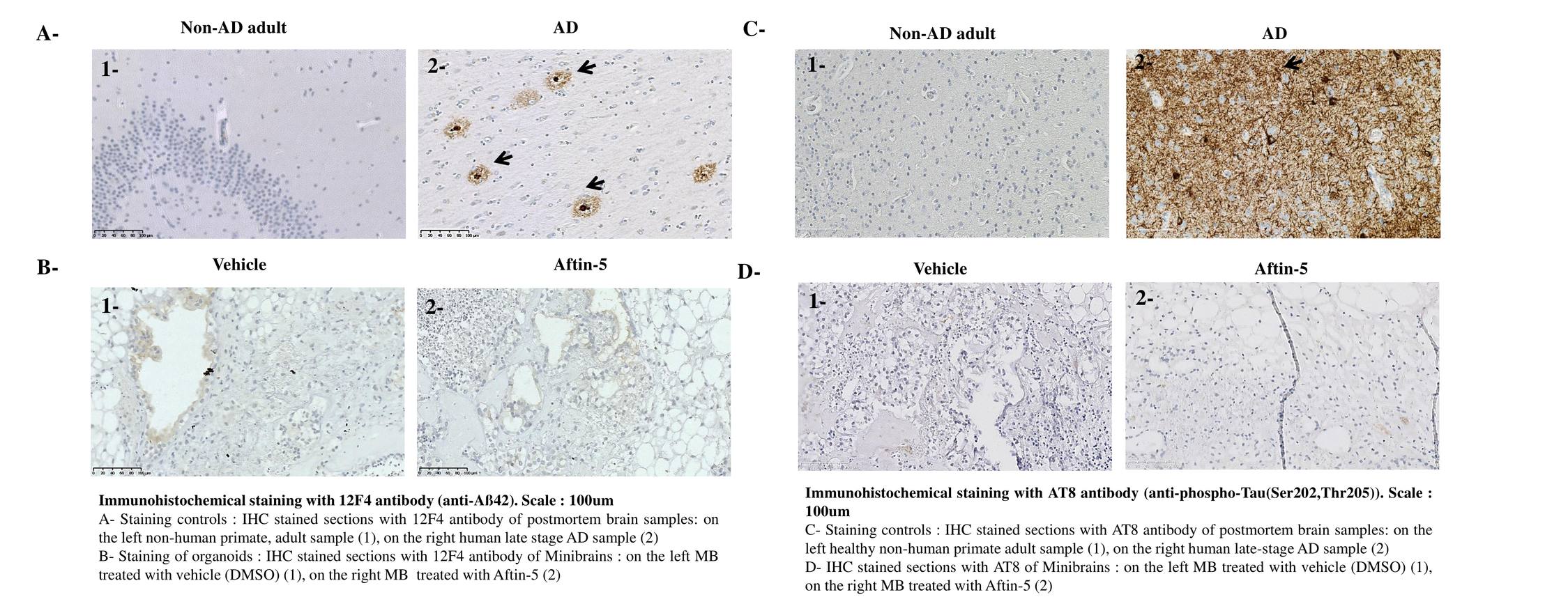

Supplement: S4 Fig — IHC with 12F4 (anti-Aß42) and AT8 (anti-phospho-Tau(Ser202,Thr205). A- Staining controls: IHC stained sections with 12F4 antibody of postmortem brain samples: on the left non-human primate, adult sample (1), on the right human late stage AD sample (2) B- Staining of organoids: IHC stained sections with 12F4 antibody of Minibrains: on the left MB treated with vehicle (DMSO) (1), on the right MB treated with Aftin-5 (2) C- Staining controls: IHC stained sections with AT8 antibody of postmortem brain samples: on the left healthy non-human primate adult sample (1), on the right human late-stage AD sample (2) D- IHC stained sections with AT8 of Minibrains: on the left MB treated with vehicle (DMSO) (1), on the right MB treated with Aftin-5 (2). Scale bars: 100 μm. (TIF) [file pone.0209150.s004.tif]

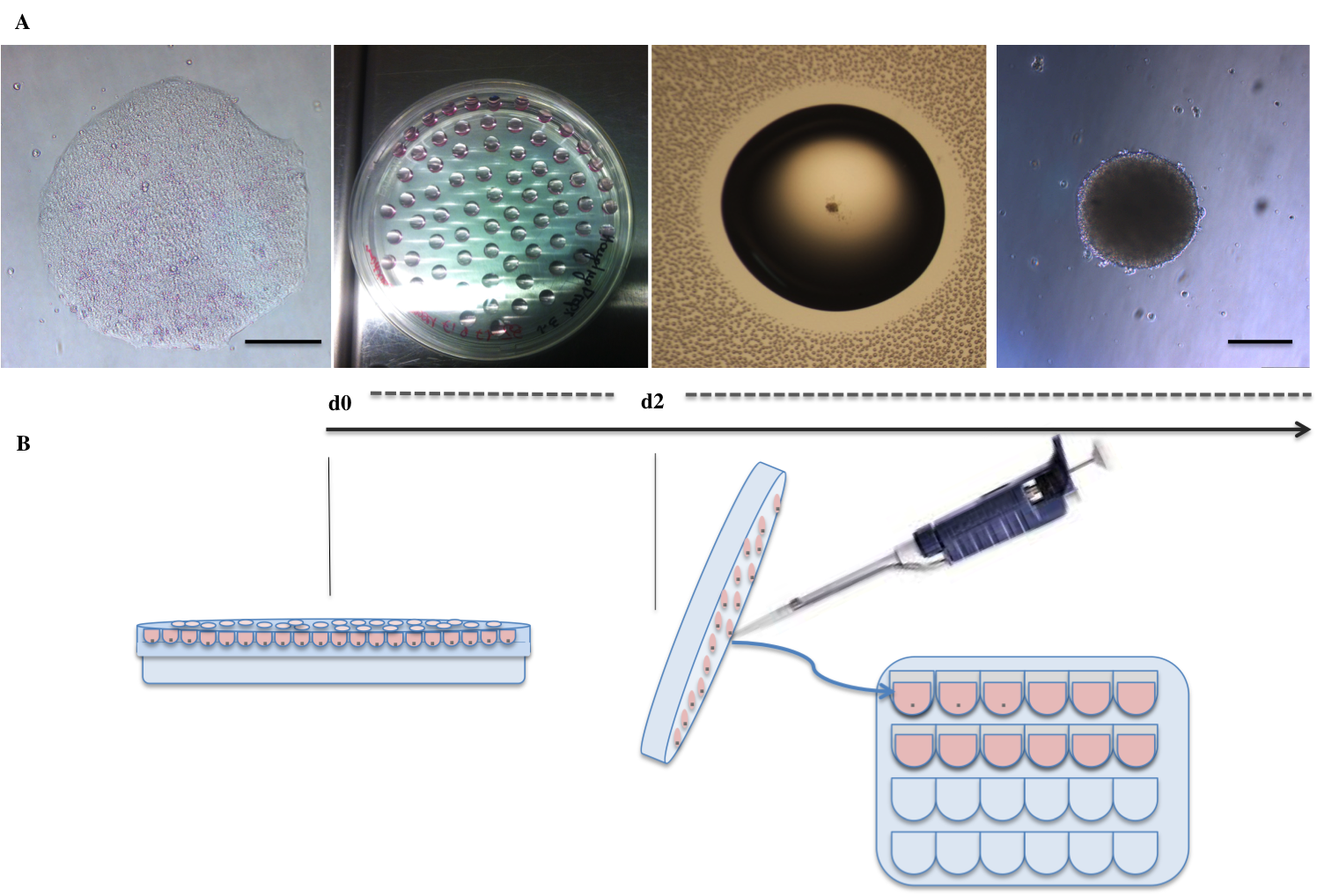

Supplement: S5 Fig — (A) iPSC colony cultured on Matrigel before dissociation (corresponding to day 0 of the MB protocol, on the left), hanging drop culture of iPS cell suspension on a petri dish cover (day 0—day 2, second picture); iPSCs were maintained in drop culture for 2 days allowing cells to aggregate, forming the EB in the center of the hanging drop and each drop contains one EB (third picture) which is harvested at day 2 (right). (B) Representation of the two step hanging drop method; EBs were cultured in the hanging drops for 2 days; then, each EB was recovered manually with a cut micropipette tip and placed into a 24-well plate containing EB medium. (TIF) [file pone.0209150.s005.tif]

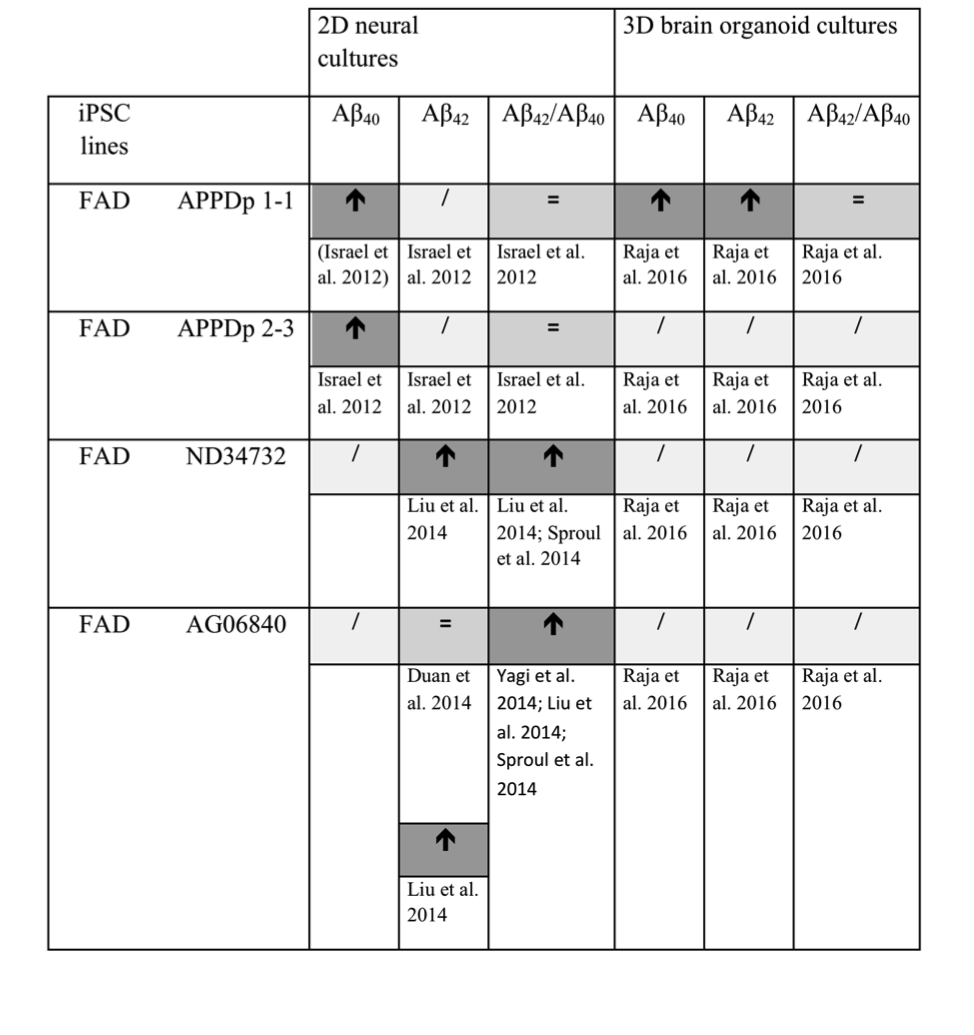

Supplement: S1 Table — In the study of Raja et al. 2016, all four FAD lines were used to create 3D brain organoids. APPDp 1–1, APPDp 2–3 (EOAD) carrying APP duplication were used in the study of Israel et al. 2012 whereas FAD lines ND34732 (PSEN1 M146I) and AG06840 (PSEN1 A264E) were used in other studies as indicated in the respective lines. Results indicated in the table refer to extracellular measures of Aβ peptide concentrations (Duan et al. 2014; Yagi et al. 2014; Liu et al. 2014; Sproul et al. 2014).(□) Increase in the concentration of Aβ compared to control lines; (=) no change in the concentration observed between FAD and control lines; (/) unknown. (TIF) [file pone.0209150.s006.tif]
